# EGF Receptor Stalls upon Activation as Evidenced by Complementary Fluorescence Correlation Spectroscopy and Fluorescence Recovery after Photobleaching Measurements

**DOI:** 10.3390/ijms20133370

**Published:** 2019-07-09

**Authors:** György Vámosi, Elza Friedländer-Brock, Shehu M. Ibrahim, Roland Brock, János Szöllősi, György Vereb

**Affiliations:** 1 Department of Biophysics and Cell Biology, Faculty of Medicine, University of Debrecen, Egyetem tér 1, H-4032 Debrecen, Hungary; 2 Max Planck Institute for Biophysical Chemistry, Laboratory of Cellular Dynamics, Am Fassberg 11, D-37077 Göttingen, Germany; 3 Department of Biochemistry, Radboud Institute for Molecular Life Sciences, Radboud University Medical Center, Geert Grooteplein 28, 6525 GA Nijmegen, The Netherlands; 4 MTA-DE Cell Biology and Signaling Research Group, Faculty of Medicine, University of Debrecen, Egyetem tér 1, H-4032 Debrecen, Hungary; 5 Faculty of Pharmacy, University of Debrecen, Egyetem tér 1, H-4032 Debrecen, Hungary

**Keywords:** Fluorescence correlation spectroscopy, FCS, fluorescence recovery after photobleaching, FRAP, epidermal growth factor receptor, translational diffusion, EGFR–eGFP fusion protein

## Abstract

To elucidate the molecular details of the activation-associated clustering of epidermal growth factor receptors (EGFRs), the time course of the mobility and aggregation states of eGFP tagged EGFR in the membranes of Chinese hamster ovary (CHO) cells was assessed by in situ mobility assays. Fluorescence correlation spectroscopy (FCS) was used to probe molecular movements of small ensembles of molecules over short distances and time scales, and to report on the state of aggregation. The diffusion of larger ensembles of molecules over longer distances (and time scales) was investigated by fluorescence recovery after photobleaching (FRAP). Autocorrelation functions could be best fitted by a two-component diffusion model corrected for triplet formation and blinking. The slow, 100–1000 ms component was attributed to membrane localized receptors moving with free Brownian diffusion, whereas the fast, ms component was assigned to cytosolic receptors or their fragments. Upon stimulation with 50 nM EGF, a significant decrease from 0.11 to 0.07 μm^2^/s in the diffusion coefficient of membrane-localized receptors was observed, followed by recovery to the original value in ~20 min. In contrast, the apparent brightness of diffusing species remained the same. Stripe FRAP experiments yielded a decrease in long-range molecular mobility directly after stimulation, evidenced by an increase in the recovery time of the slow component from 13 to 21.9 s. Our observations are best explained by the transient attachment of ligand-bound EGFRs to immobile or slowly moving structures such as the cytoskeleton or large, previously photobleached receptor aggregates.

## 1. Introduction

The epidermal growth factor receptor (EGFR) is a member of the ErbB receptor family [1]. The transmembrane protein contains an extracellular ligand binding domain, a single hydrophobic transmembrane domain, and an intracellular, highly conserved tyrosine kinase unit. Although it is generally accepted that a receptor dimer in complex with a ligand is responsible for inducing the signal transduction cascade [2,3,4], and also a crystal structure of the ligand bound receptor is available [5], the exact mechanism of EGFR activation and the impact it has on receptor organization in the plasma membrane are still under debate. For recent reviews on EGFR conformation and signaling, see [6,7,8]. Essentially, two models have been proposed.

According to the model of ligand-induced dimerization, EGF receptors are initially monomeric [9,10,11], and their intrinsically active kinase domains are separated from each other in the absence of stimulation. This model is supported, among others, by the following observations. Upon incubation of cells with EGF, oligomerization was detected by electron microscopy of immunogold-labeled EGFR [12]. Titration calorimetry and small-angle X-ray scattering led to the formulation of a model, in which the extracellular domain of EGFR dimerizes only after binding to an EGF molecule [13].

The conformational activation model [14] assumes preformed dimers. Ligand binding increases kinase activity by inducing a conformational change in the transmembrane segment. This model is supported by FRET data [14] as well as data from Stern and coworkers [15], demonstrating that a point mutation in the transmembrane domain can enhance kinase activity even without the binding of EGF. It has been shown by chemical crosslinking followed by sucrose gradient centrifugation that EGFR has the ability to form dimers, even in the absence of a ligand [16], and the authors also presented evidence for EGF inducing a twist in the juxtamembrane domain of the receptor upon binding. 

Quantitative analyses of receptor dimerization by biophysical techniques all provided support for a capacity of the receptor to dimerize in the unliganded state. Photon counting histogram analysis showed that next to a majority of unstimulated EGFR–eGFP molecules expressed in Chinese hamster ovary (CHO) cells in a monomeric state (~70%), ~20% formed dimers and 10% formed oligomers [17]. Lipid raft disruption by methyl-β-cyclodextrane-mediated cholesterol depletion increased, whereas cholesterol enrichment decreased the tendency for oligomerization. NMR studies and molecular dynamics simulations revealed that the dimerization of EGFR is influenced by the local lipid environment of the transmembrane domain of the receptor [18]. Using solid state NMR, Kaplan et al. showed that in unstimulated EGFRs the extracellular domain is highly dynamic, while the intracellular kinase domain is rigid. The binding of EGF then restricts the overall and local motion of EGFR domains [19].

Nagy et al. used number and brightness analysis to characterize the aggregation state of erbB proteins. They found EGFR–eGFP in CHO cells to be predominantly monomeric in low expressers (up to 200,000 receptors), whereas in high expressers (>500,000), 30% were present as preformed dimers. EGF induced dimerization/oligomerization and colocalization with clathrin-coated pits [20]. It must be noted that number and brightness analysis, similarly to fluorescence correlation spectroscopy (FCS), is a fluctuation method and is therefore blind to immobile structures such as cytoskeleton-bound receptor aggregates.

In addition to fluctuation-based methods, fluorescence recovery after photobleaching (FRAP) [21] or single particle tracking [22] have also been successfully employed for measuring receptor mobility. Using dual-color single particle tracking, it was also shown that unliganded EGF receptors can transiently co-diffuse [23], and co-confined receptors can be stabilized as dimers by ligand binding [24]. Such transient association can readily explain a concentration dependence of steady-state receptor dimer levels. 

In this paper, fluorescence correlation spectroscopy (FCS) and fluorescence recovery after photobleaching (FRAP) were employed to investigate activation-induced changes in receptor mobility and aggregation at different time and distance scales. Chinese hamster ovary (CHO) cells stably transfected with an EGFR–eGFP fusion protein served as a model system. We show that the two techniques arrive at complementary and coherent descriptions of receptor organization on the submicrometer as well as the micrometer scales. The data show a transient slowing down of the mobile fraction of EGFR upon activation, with no apparent change in the aggregate stoichiometry of this receptor pool.

## 2. Results

### 2.1. Measurement and Analysis of Autocorrelation Functions

**Figure 1:**
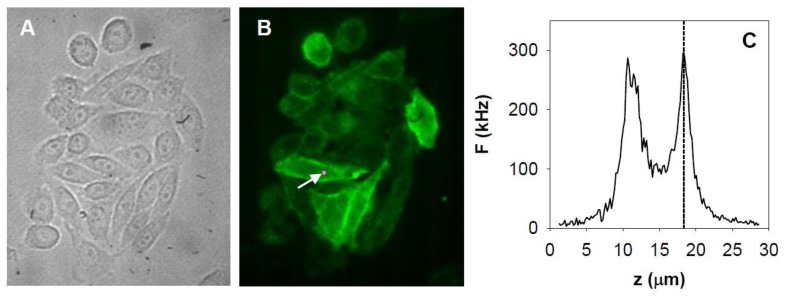
Microscopic images of epidermal growth factor receptor (EGFR)–eGFP expressing cells. (**A**) Transmission and (**B**) fluorescence images of Chinese hamster ovary (CHO) F1-10 cells expressing EGFR–eGFP. The spot at the arrowhead indicates the focal volume during a fluorescence correlation spectroscopy (FCS) measurement. (**C**) Fluorescence intensity profile across the cell. Fluorescence intensity vs. objective position was detected as the piezoelectric focus positioner moved the objective towards the sample. The dashed line indicates the upper membrane (distal from the cover slip), where FCS measurements were performed.

We used fluorescence correlation spectroscopy to determine the local diffusion coefficients, diffusion mechanism, concentration and apparent brightness reflecting the state of aggregation of EGFR–eGFP in live CHO cells. Adherent, elongated, dimly fluorescent cells were selected for measurements; the average number of EGFR–eGFP molecules in the femtoliter-sized detection volume (Figure 1A,B) was in the range of ~0.1 to ~40. Measurements were carried out in the upper (apical) plasma membrane (Figure 1C) over 60–200 s. 

**Figure 2:**
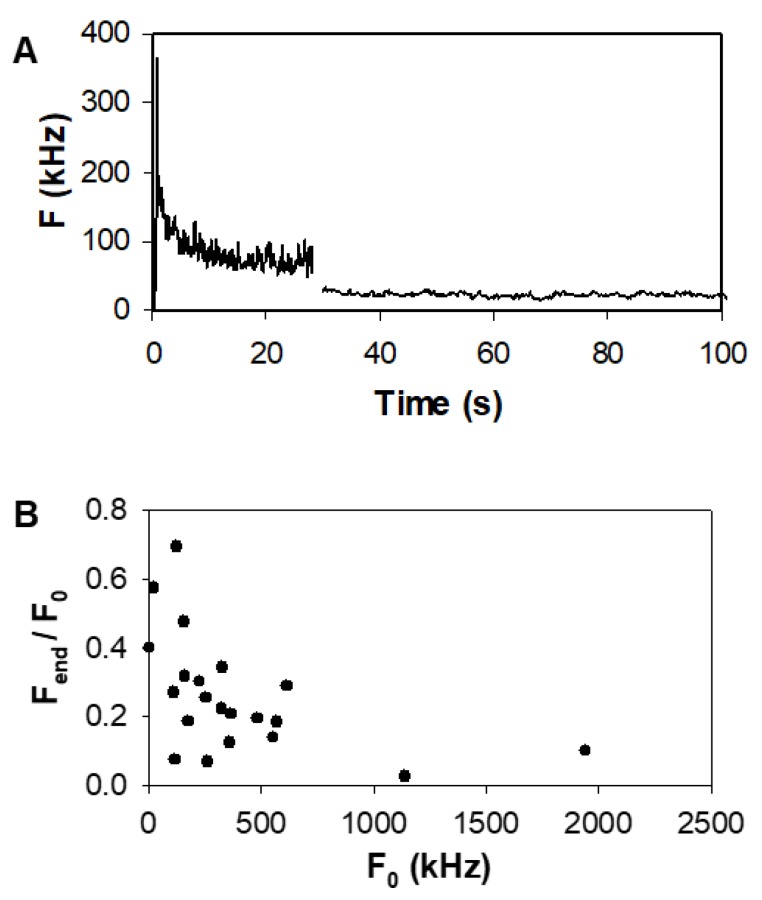
Correlation between fluorescence intensity and the immobile fraction of EGFR–eGFP (**A**) Pre-bleaching of low-mobility and immobile EGFR–eGFPs. During the 30-s bleaching period, the laser power was 9–10 kW/cm^2^; then, for the FCS measurement, it was reduced to 0.9 kW/cm^2^. (**B**) Final fluorescence intensity F_end_ measured after a 30 s illumination, normalized to the initial intensity F_0_, plotted as a function of F_0_. Illumination power density was 9 kW/cm^2^. The negative correlation of the ratio with expression level indicates that the fraction of immobile receptors is larger in high expressers.

In order to bleach low-mobility or immobile molecules prior to the measurement, the laser was switched on at 9–10 kW/cm^2^ until a steady signal was reached (at about 30 s, Figure 2A). To characterize the fraction of bleached receptors, the final fluorescence intensity *F_end_*, normalized to the initial intensity *F*_0_, was plotted as *F_end_/F*_0_ vs. *F*_0_ (Figure 2B).

**Figure 3:**
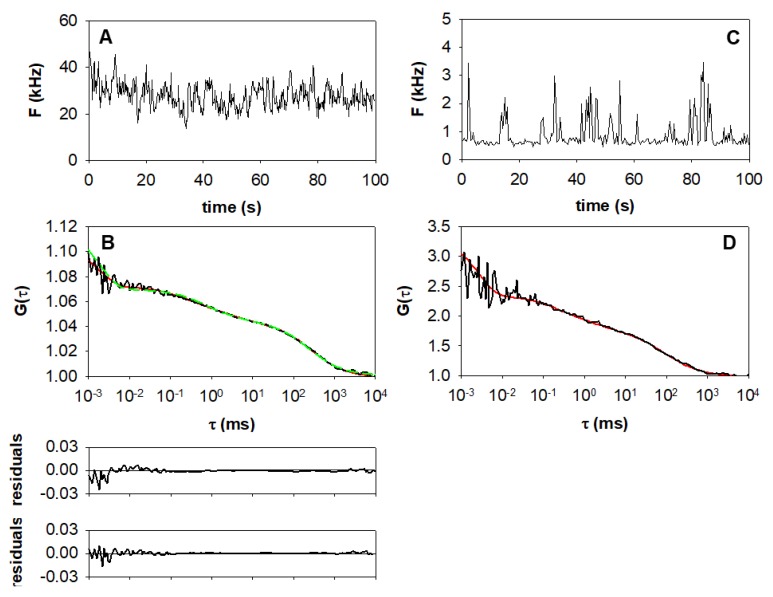
Autocorrelation curves fitted with models containing two diffusing components (**A**) Count trace recorded during an FCS measurement on a resting F1-10 cell. The pre-bleached sample has a steady running average of fluorescence intensity. (**B**) Autocorrelation curve (black) and nonlinear fits. Model 1 (green dashed line) assumes a triplet state and two diffusing components with free Brownian diffusion. Model 2 (red solid line) considers a triplet state formation, EGFP blinking and two diffusing components, also allowing obstructed diffusion. Residuals are shown below for model 1 (top) and model 2 (bottom). (**C**) Count trace recorded from an F1-10 cell with a very low expression level. Background intensity was assessed to be ~0.5 kHz. (**D**) Autocorrelation curve and nonlinear fit according to model 2. The high amplitude refers to a low concentration: the mean number of EGFR–eGFP molecules in the detection volume was ~0.12.

**Figure A1:**
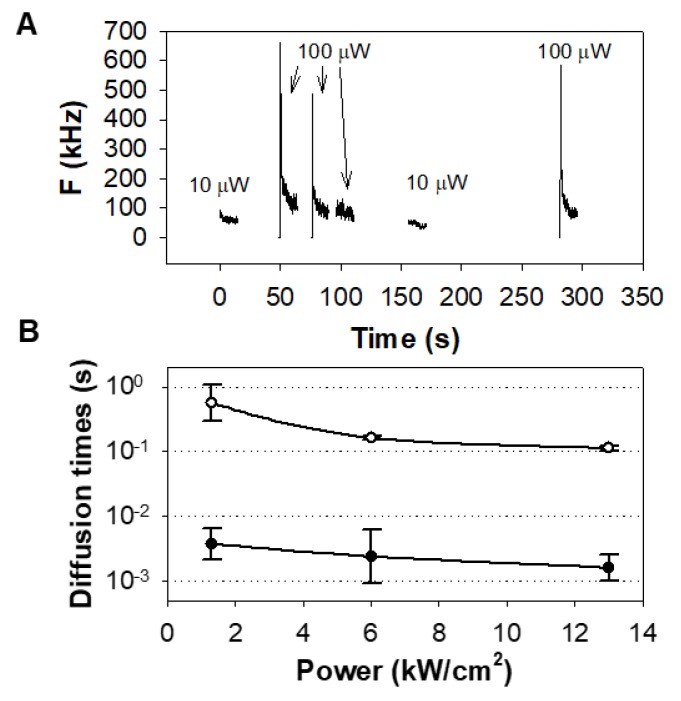
Effect of illumination intensity on the fluorescence intensity and the apparent diffusion time. (**A**) Count traces during repetitive bleaching and monitoring intervals. Count rate was monitored using 0.9 kW/cm^2^ illumination power density at the sample, while bleaching was done with 9 kW/cm^2^. (**B**) Dependence of slow (open circles) and fast (filled circles) diffusion times on illumination power.

The bleached fraction increased with increasing initial intensity, indicating that the fraction of low-mobility/immobile molecules was larger in high expressers. If using intermittent illumination at 10 kW/cm^2^, fluorescence recovered close to its original value over 150–200 s, indicating that the photobleached receptors were not completely immobile (see Appendix A, Figure A1A). We illuminated the sample at the selected spot for 30 s with 10 kW/cm^2^ light density to bleach immobile/slowly moving aggregates, which would have compromised the autocorrelation curves. This illumination may also have resulted in a light-driven transition of eGFP to a dark state. After the 30 s pre-bleach period, several consecutive autocorrelation measurements were recorded at 1 kW/cm^2^. The autocorrelation functions acquired from EGFR–eGFP expressing cells required multiple components for fitting, implying that fluctuations resulting from several photophysical and diffusion processes were present simultaneously (Figure 3). 

Several different models were tested. The simplest model reasonably fitting the autocorrelation functions assumed a triplet component and two species diffusing by free Brownian diffusion (Equations (1) and (5)). The triplet component was in the microsecond domain (*τ_tr_* = 2.0 ± 0.8 μs). The decay of the autocorrelation function in the lower millisecond domain was attributed to a fast diffusion process, likely resulting from cytosolic EGFR–eGFP or its fragments [25]. The slow diffusion time was attributed to the diffusion of EGFR–eGFP fusion proteins in the plasma membrane.

In spite of using a stably transfected cell line, the expression levels of EGFR–eGFP had a rather broad distribution. In some cases, the number of molecules in the detection volume was significantly smaller than one (Figure 3C,D), where the fluctuation traces showed distinct molecules or aggregates entering and exiting the detection volume. In these cases, correction for uncorrelated background was indispensable for the determination of *N* (Equation (9)). The background was derived from the baseline of the count traces. With a detection volume of 1.46 fL (1.46 μm^3^), such a low number of molecules (*N*~0.12 in the confocal volume) corresponded to a receptor concentration as low as 0.33 particles per μm^2^.

In a number of cases, the model with two distinct freely diffusing components failed to describe the data accurately in the sub-millisecond range (Figure 3B, green fit trace and first residual plot). Therefore, we used a model (Equations (2) and (6)) also assuming eGFP blinking [26], yielding a mean correlation time of ~180–190 μs. This model also included anomalous diffusion [27,28] of both diffusing species. The anomaly parameter α was used to express the degree of non-linearity of the root mean squared displacement vs. time function. For free Brownian diffusion (model 1) α = 1, for obstructed diffusion α < 1, and the slope of the autocorrelation function around the diffusion time is smaller than in the case of free diffusion [29]. The fit parameters obtained for models 1 and 2 are summarized in Table 1. The averaged mean squared error (mse) of the fits for model 2 is slightly lower (3.5 vs. 3.9), indicating that it gives a better account of the experimental data. Diffusion coefficients were calculated from the diffusion times according to Equation (12). In unstimulated cells, the value of the anomaly parameter was close to 1 for both components, indicating that only little obstruction of free diffusion occurred.

### 2.2. FCS Measurements on EGF-Stimulated Cells

**Figure 4:**
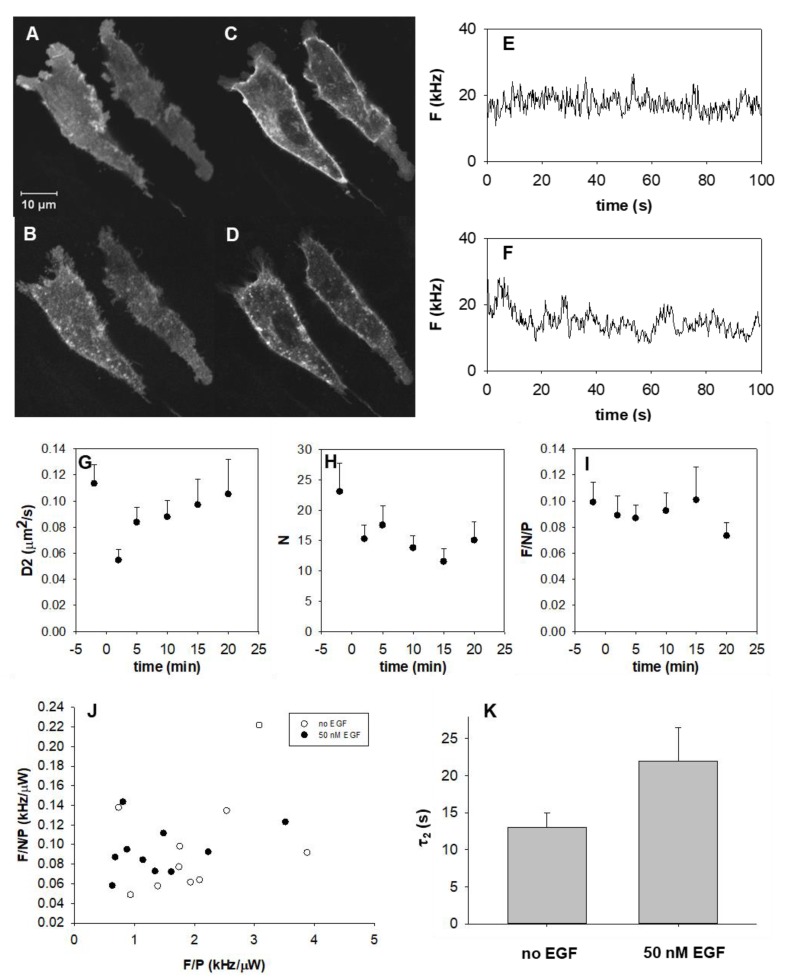
Effect of EGF stimulation on the dynamics of EGFR–eGFP. (**A**–**D**) Confocal sections of F1-10 cells taken before (**A**,**C**) and ~15 min after EGF stimulation (**B**,**D**). In resting cells, the distribution of receptors is more uniform than in stimulated cells, in which it is more granulated. Images (**A**) and (**B**) were recorded in the plane of the plasma membrane, whereas (**C**) and (**D**) were recorded 1 mm above this plane in the cytoplasm. (**E**,**F**) Typical fluorescence fluctuation recorded before, and 2 min after 50 nM EGF stimulation. (**G**–**I**) Time dependence of the slow diffusion coefficient, *D*_2_, of the apparent number, *N*, of independently moving objects in the detection volume, and of the normalized specific fluorescence per particle *F*/*N*/*P* (molecular brightness), with 50 nM EGF added at *t* = 0 min. *F* is the fluorescence intensity in kHz, *N* is the average number of molecules in the detection volume and *P* is the laser power. Mean ± SEM of >10 independent measurements. (**J**) Molecular brightness does not vary with increasing expression level *F*/*P*. (**K**) Recovery time of slow component (with 95% confidence interval) from biexponential fit determined from stripe photobleaching FRAP experiments before and after stimulation with 50 nM EGF (*n* = 7).

**Figure A2:**
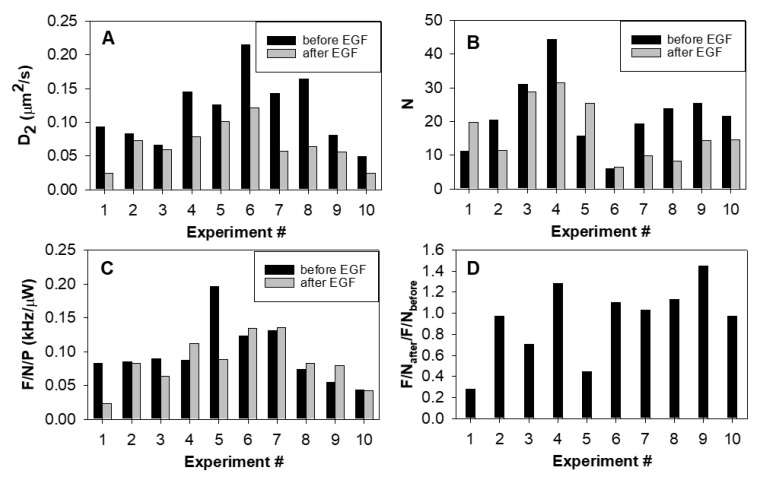
Effect of EGF stimulation on the dynamics of EGFR–eGFP in individual F1-10 cells. Black bars: before treatment, grey bars: after treatment. (**A**) Change of the slow diffusion coefficient, *D*_2_, upon stimulation by 50 nM EGF. (**B**) Change of the apparent number, *N*, of independently moving particles in the detection volume. (**C**) Change of the normalized specific fluorescence per molecule (specific brightness). *F* is the fluorescence intensity in kHz, *N* is the average number of molecules in the detection volume and *P* is the laser power. (**D**) Ratio of specific brightness values after stimulation to values before stimulation. Apart from a few outliers, the value is around 1.

In resting cells, the EGFR–eGFP fusion proteins showed a relatively uniform lateral distribution (Figure 4A,C). Stimulation with EGF led to receptor aggregation and internalization within 5 min, which was still apparent even 15–30 min after stimulation (Figure 4B,D). Stimulation was followed by autocorrelation measurements at the same spot where pre-stimulation measurements had been recorded. Average parameters derived from autocorrelation measurements in the same membrane spot acquired directly before and after adding 50 nM EGF to the cells are shown in Table 1 (parameters for ten individual cells are presented in the Appendix A, Figure A2). 

Fluorescence fluctuations became slower (Figure 4E,F) and, correspondingly, the slow, membrane-related diffusion coefficient *D*_2_ decreased ~1.8 fold directly (~2 min) after EGF stimulation, according to model 2 (Table 1, Figure 4G). The slowing down of receptor mobility was transient: over the following 20 min, *D*_2_ recovered to near to its pre-stimulation value. At the same time, the number of independently moving objects *N* in the detection volume dropped to ~78% of the initial value, and remained low during the whole observation period (Figure 4H). Stimulation had no impact on the anomaly parameter *α*_2_ (Table 1), which means that also after activation the diffusion process in the membrane could be approximated by free Brownian motion. For the fast component, *α*_1_ decreased from 0.94 to 0.82.

The extent of receptor aggregation was further addressed by calculating the apparent brightness, which is the fluorescence per molecule (diffusing entity) normalized to the laser power. The apparent brightness is obtained by dividing the background-corrected mean fluorescence *F* by the background-corrected apparent number of molecules *N_app_* and by the laser power *P* as $fpm=FN_{app}^{-1}P^{-1}$. The mean fluorescence *F* decreased after stimulation, but only to the same extent as the number of particles *N_app_*. Consequently, the mean *fpm* was unaffected: the average ratio of the *fpm* value after and before stimulation was close to 1 (0.94 ± 0.36) (Figure 4I, Figure A2C,D). This implies that unbleached mobile receptors did not aggregate with each other upon stimulation. We found no correlation between *fpm* and *F*/*P* neither for unstimulated nor for stimulated cells, implying that the clustering of mobile receptors did not depend on receptor density in the low expression range studied by FCS (Figure 4J). On the other hand, the increased amount of bleached receptors at higher expression levels (Figure 2B) suggests that a higher expression level is associated with a higher fraction of low-mobility receptors.

### 2.3. FRAP Measurements

Fluorescence recovery after photobleaching (FRAP) is a classic technique to measure the average mobility of a large ensemble of molecules in cell membranes and other organelles. The mobility of EGFR–eGFP on resting and ligand-activated cells was first characterized by the half recovery time *T*_1/2_, which is the time point corresponding to the midpoint of fluorescence intensity between the value measured directly after the bleaching pulse and the final stabilized value (~50 s later). We found a significant increase in the *T*_1/2_ value from 2.7 to 6.4 s, by a factor of about 2.4 upon stimulation. This change is in good agreement with the factor 2 decrease in diffusion constants, derived from the FCS measurements. These data imply that, similarly to the FCS-determined shorter-range mobility over a few hundred nm (e^−2^ radius of FCS detection efficiency: 0.34 µm), longer-range mobility in the 1 µm range probed by FRAP (width of bleached area: 1.1 µm) also decreases upon ligand binding. 

**Figure A3:**
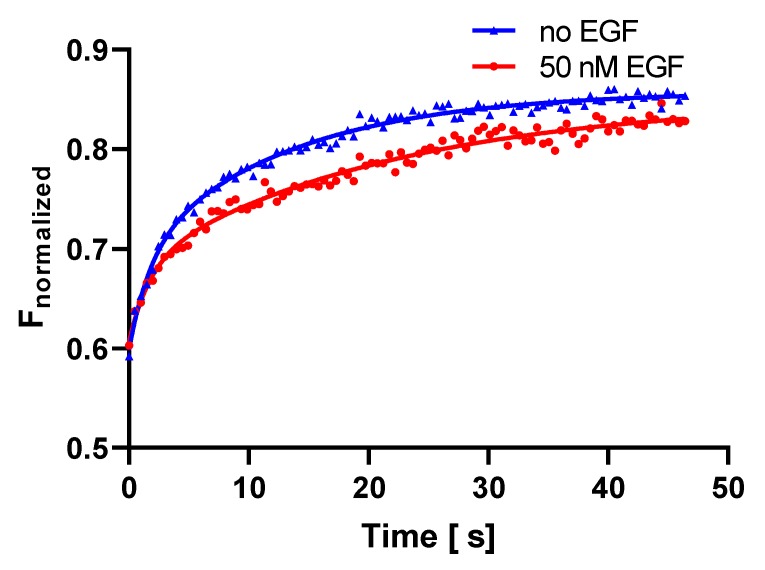
Average fluorescence recovery after photobleaching curves measured with F1-10 cells. The fluorescence intensity of EGFR–GFP was excited with 1 kW/cm^2^ laser power (488 nm). Bleaching was achieved by scanning a 10 pixel (1.1 μm) wide stripe across the shorter axis of the cell with 50 kW/cm^2^ laser power. Fluorescence intensity of the bleached region-of-interest (ROI) was divided by the average fluorescence intensity of the whole cell at each time point, and was normalized to the pre-bleach intensities. Measurements were carried out before and ~2.5 min after stimulation with 50 nM EGF. The average recovery curves of seven untreated and eight EGF-treated cells are shown. The solid line represents a biexponential fit with a fast decay time of 1.7 s (shared by the two curves) and slow decay times of 13 s and 21.9 s for the control and EGF-treated samples.

Analysis was also carried out in a more sophisticated way (Figure 4K, Figure A3). Fluorescence intensity was corrected for the overall bleaching of the cell by dividing the intensity measured in the bleached region-of-interest (ROI) by the average intensity measured from the whole cell (Figure A3). A biexponential fit of the normalized, averaged recovery curves yielded a fast component with a decay time of *τ*_1_~1.7 s shared by control and EGF-treated cells (fast fractions: 0.36 and 0.31), which may be due to cytoplasmic diffusion or to recovery from a light-induced long dark state. These values are rough estimates due to the relatively low frame rate (2 frames/s). However, they coincide well with the estimated off-time, 1.6 s, of light-induced dark states as assessed from the fluorescence recovery curves of immobilized eGFP-tagged histones (manuscript in preparation). The decay time of the slow component increased by a factor of 1.7 upon EGF treatment (from *τ*_2_~13.0 s to 21.9 s, Figure 4K); this component can be attributed to diffusion in the plasma membrane. If we convert *τ*_2_ values to half-lives (multiplying by ln(2)), we obtain 9.0 and 15.2 s, which yield *D* values of 0.067 and 0.040 µm^2^/s when substituted into the Einstein–Smoluchowski equation ($D=\Delta x^{2}/\left(2T_{1/2}\right)$). These are similar to the FCS-derived slow diffusion coefficients (Table 1). Using this analysis, the immobile fractions were 0.14 and 0.15 for control and EGF-treated cells, respectively.

### 2.4. Signaling Competence of EGFR–eGFP

**Figure 5:**
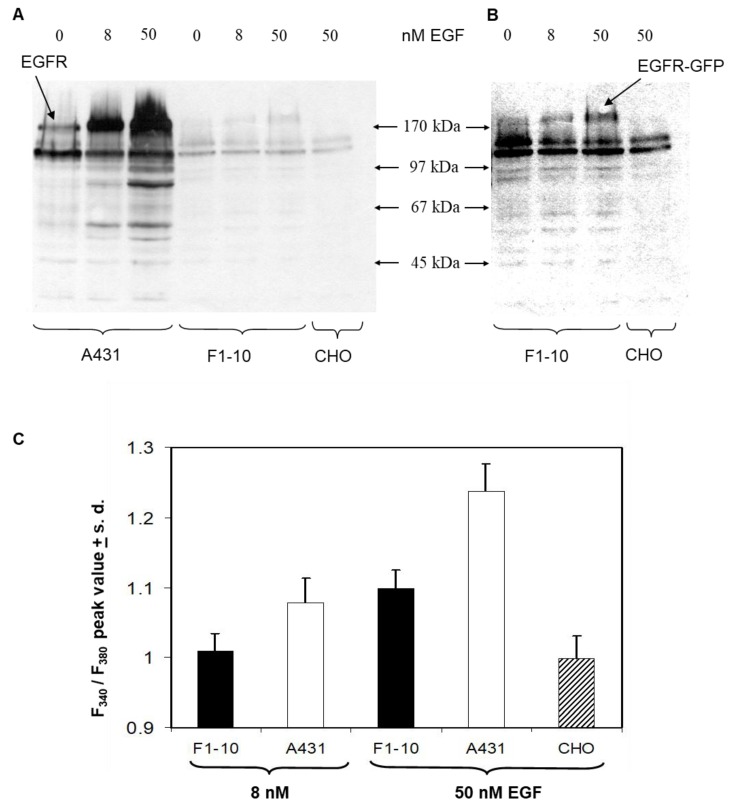
Signaling competence of EGFR–eGFP. (**A**,**B**) Western-blot of EGFR in A431, F1-10 and CHO cells treated with different concentrations of EGF. Panel (**B**) is a contrast enhanced version of the weaker lanes from F1-10 and CHO cells. (**C**) EGF-induced calcium response in F1-10 and A431 cells measured with Fura-2 Ca^2+^-indicator.

The signaling competence of EGFR–eGFP fusion proteins was assessed by analysis of ligand-induced tyrosine phosphorylation of cellular proteins and detection of calcium signaling upon receptor stimulation with EGF (Figure 5). Previously, the capacity of the EGFR–eGFP fusion proteins to bind ligands and to internalize was assessed by fluorescence microscopy [25,30]. Signal transduction through the EGFR is initiated by induction of proximity of the tyrosine kinase domains and subsequent trans-phosphorylation of tyrosine residues in the receptor C-terminal domain and downstream substrates. Lysates of ligand-treated and control cells were probed for tyrosine phosphorylated proteins by Western blot (Figure 5A,B). 

The human epidermoid carcinoma cell line A431 served as a positive control. In A431 cells, as well as F1-10 cells, the ligand-induced tyrosine phosphorylation of bands with ~170–190 kDa molecular mass, corresponding to the EGFR, was dose-dependent. Due to its larger molecular mass, the mobility of the EGFR–eGFP fusion protein was lower than that of the native EGF receptor. Other proteins at ~60, 65, 90 and 100 kDa were also phosphorylated in a dose-dependent manner. In non-transfected CHO cells, only two weak bands with 100 and 110 kDa molecular mass were present, and although these were also present in A431 and F1-10 cells, their phosphorylation state did not change upon EGF treatment. On average, the F1-10 cell line expresses only 2 × 10^4^ copies of the EGFR–eGFP fusion protein compared to 1–2 × 10^6^ EGFRs in A431 cells as determined in our lab by Qifikit; for A431 cells, an average copy number of 1.2 × 10^6^ was found by [31]. For this reason, the intensity of the band corresponding to the receptor was much weaker in F1-10 than in A431 cells.

Downstream signaling was further monitored by measuring the EGF-induced Ca^2+^-response. Phospholipase C is one of the early downstream effectors of EGF receptor signaling. The activation of this enzyme by binding to phosphotyrosine residues of EGFR leads to the generation of diacylglycerol and the release of inositol-trisphosphate, eliciting an increase in intracellular calcium [32]. The aim of the Ca^2+^ measurements was for the demonstration of Ca^2+^ responses and illustration of their relative magnitude for different cells and different conditions. Changes in intracellular calcium were detected by ratiometric imaging of the fluorescence of the Ca-indicator dye Fura-2 excited at 340 and 380 nm (Figure 5C). For both A431 cells and F1-10 cells, stimulation with 50 nM EGF led to an increase in intracellular calcium. At 8 nM EGF, a calcium response was detectable in A431 cells, only. CHO negative controls did not exhibit an increase in intracellular calcium at any EGF concentration.

## 3. Discussion

We used two complementary methods to follow the dynamics of EGFR diffusion at time scales of a few hundred ms to a few seconds and distance scales of a few hundred nm to around a µm in resting and stimulated cells. FCS probes the shorter-range diffusion of small ensembles of mobile receptors, whereas FRAP informs about longer-range average diffusion of molecular ensembles. FCS is confined to studying only mobile receptors, while FRAP provides information about the whole receptor population, including immobile entities. Both these techniques indicated—indirectly or directly—the presence of a significant fraction of low-mobility or immobile molecules and the decrease of the diffusion coefficient of the mobile fraction upon ligand stimulation. The possible scenarios causing receptor slow-down are discussed below.

### 3.1. Effect of Bleaching/Light-Induced Blinking on Autocorrelation Measurements

In all cells, a strong decrease in fluorescence was observed in the initial seconds of illumination. Recovery of fluorescence in the absence of laser excitation indicated that this decaying signal originated from the bleaching of a fraction of receptors with little mobility and/or from a light-induced isomerization process leading to a transient nonfluorescent state [33,34,35]. The bleached fraction was larger for higher expressers (Figure 2B), which refers to a higher fraction with very low mobility according to continuous photobleaching theory [36]. This observation provides evidence that light-induced isomerization alone cannot fully explain the initial decrease in fluorescence. 

Unfortunately, the lowering of laser power was no solution to this problem. When the laser power was reduced to a level at which bleaching could be almost completely avoided, the time required to obtain an autocorrelation curve increased, resulting in a dominance of movements of the plasma membrane in the autocorrelation function (not shown). Apparently, one fraction of receptors had a diffusion rate too low to be distinguished from cellular movements by FCS, while a second fraction with higher mobility could be distinguished. For this reason, the fraction of receptors with little mobility was pre-bleached at higher excitation intensities and autocorrelation functions were recorded at a lower laser power. On one hand, this protocol eliminated a continuous loss of intensity during FCS measurements due to the depletion of the low-mobility fraction. On the other hand, it confined the quantification of receptor numbers and diffusion to the fraction of receptors with higher mobility. The large fraction of receptors with low mobility defines a practical limitation of FCS in cellular studies set forth by the sensitivity of the method to the displacement of the membrane with respect to the detection volume. This prevents measurements of diffusional autocorrelation times around or above a few seconds (in our case, diffusion times longer than a few seconds). For investigating such slow diffusion processes, FRAP is the more adequate approach.

After several minutes of laser illumination at a single spot in the membrane using the typical power density applied in our FCS measurements (~1 kW/cm^2^), the extent of overall depletion of fluorescence in the cell derived from integrating all pixel-intensities was about 20%. This is larger than the percentage of the illuminated area out of the total cell surface area, implying that not only those receptors located in the membrane domain initially overlapping with the focal volume were bleached, but receptors from more distant membrane regions could reach the illuminated area within the time span of the measurements. This observation evidences the long-range diffusion of the receptors in the plasma membrane.

### 3.2. Effects of Receptor Stimulation on Autocorrelation Measurements

After the stimulation of receptors with EGF, a significant but transient decrease in receptor mobility was observed. This decrease coincided with a reduction in the number of molecules suggesting that, following stimulation, receptors were cleared from the cell surface. The decrease in the diffusion constant was independent of the model used for the fit. This finding illustrates the robustness of the evaluation of slow (intramembrane) diffusion times. However, the two-fold decrease in *D* strongly exceeds the theoretically predictable value for dimerization. This discrepancy hints at the (transient) interaction of EGFR with static structures such as the underlying cytoskeleton [37]. A higher order oligomerization of the receptors could also explain the drastic decrease in *D*, but this hypothesis is inconsistent with the meek 22% decrease in the number of independently diffusing species. The constancy of the normalized fluorescence per molecule also indicates that, at least in the first few minutes of activation, the aggregate size of mobile EGFRs did not change significantly.

However, we have to keep in mind that the chromophore of a large fraction of the receptors is bleached and therefore does not contribute to the signal in FCS. Also, the efficiency of chromophore formation is smaller than unity [38]. Thus, it is equally plausible that receptor aggregation occurs [39], but it is only reflected by the decrease in diffusion constant as the detected EGFR–eGFP molecules or preformed dimers adhere to the more slowly moving higher order clusters that have already been photobleached in the pre-bleach period, and thus do not contribute to the specific fluorescence per particle value. This is coherent with earlier image correlation spectroscopy data, indicating that the majority of active receptors are in larger clusters [40], as well as with the finding that the Grb2 adapter is preferentially associated with tetramers of the EGFR [41]. Furthermore, using single molecule analysis, Huang et al. observed stepwise photobleaching events for EGFR in the presence of EGF, which could be best explained by EGFR multimerization occurring through the self-association of ligand-bound dimers after EGF binding [39].

According to Wohland’s group [42,43], the majority, ~68%, of EGFRs existed as preformed dimers in unstimulated CHO cells based on single wavelength FCCS. The extent of crosscorrelation increased upon stimulation with EGF from ~10 min onward, suggesting the onset of oligomerization. FCS curves were fitted with a single component model, yielding an average diffusion coefficient of 0.38 µm^2^/s, which is between our slow and fast FCS components. The same group found that both the cytoskeleton and lipid microdomains influenced the organization, mobility and ligand-induced internalization of receptors [44].

Stimulation by EGF resulted in a decrease in the anomaly parameter α_1_ and a slowing down of the diffusion of the fast component assigned to the cytoplasmic diffusion of EGFR–eGFP fragments. Rijken and coworkers found that EGF stimulation induced actin polymerization in A431 cells [45,46]; a more densely packed actin network might be responsible for the slowing down and the hindered, anomalous behavior of the diffusion of the fast, cytoplasmic component. However, we also acknowledge that the value smaller than one may also be a consequence of an incomplete overlap of the confocal detection volume with the cytoplasm.

### 3.3. FRAP Analysis Indicates Slowing Down of Long-Range Diffusion

We used FRAP to define the long-range mobility of EGFR using strip photobleaching of a larger area of the plasma membrane in resting and stimulated cells. The half recovery time increased by a factor of 2.4, whereas the slow diffusion coefficient of the biexponential fit decreased by a factor of 1.7 upon EGF stimulation, similar to the 1.8-fold decrease in diffusion coefficient derived by FCS. This change in mobility again exceeds that expected for receptor dimerization. FRAP corroborates the finding that EGF stimulation leads to transient interactions with low-mobility structures. This is coherent with the finding that a large fraction of tyrosine-phosphorylated EGFR upon ligand binding is located in clathrin-coated pits [47]. Consequently, the decrease in receptor mobility may be due to transient binding to immobile structures, such as the cytoskeleton or large, previously photobleached, less mobile receptor clusters; or due to sequestration in clathrin-coated pits.

## 4. Materials and Methods

### 4.1. Cell Culture

A431 epidermoid carcinoma cells, Chinese hamster ovary (CHO) cells and transfected CHO cells stably expressing the fusion protein of the epidermal growth factor receptor and the F64L, S65T mutant of GFP (eGFP) [25] were grown in a 5% CO_2_ humidified atmosphere at 37 °C in DMEM supplemented with 10% FCS. The subclone F1-10 used in this study expressed an estimated 2 × 10^4^ receptors per cell on average, as determined using the clone mAb 528 and Qifikit (DAKO / Agilent, Santa Clara, CA, USA) according to the manufacturer’s instructions. For EGF stimulation, 8 or 50 nM murine EGF (IC Chemikalien, Ismaning, Germany) was used. Prior to FCS stimulation, cells were kept in serum-free DMEM for 2 h to secure an EGF-free environment.

### 4.2. Fluorescence Correlation Spectroscopy: Measurement of Fluorescence Autocorrelations

For the measurement of fluorescence autocorrelations, the instrument described in [25,48] was used. Cells were grown on 12 mm diameter round coverslips in DMEM, washed twice in HBSS with 0.1% BSA, and allowed to equilibrate at 25 °C for 30 min. FCS measurements were performed using a Zeiss Axiovert 35 inverted microscope equipped with a water immersion objective (C-Apochromat 40×, NA 1.2, Carl Zeiss, Göttingen, Germany). Cells were visualized by a CCD camera in transmission and wide-field fluorescence mode (Figure 1A). The selected cell was moved into the laser focus by a motorized x-y stage (Märzhäuser, Wetzlar, Germany). The plane of the cell membrane was selected by a Pifoc piezoelectric lens positioner (Physik Instrumente, Waldbronn, Germany) (Figure 1B). For excitation, the 488 nm line of an Ar ion laser (2313-150MLYV, Uniphase, Eching, Germany) at laser power densities of ~1 kW/cm^2^ was used. Emission was detected through a 515–545 nm bandpass filter. In cells expressing the EGFR–eGFP fusion protein, the plasma membrane was apparent as peaks proximal and distal to the coverslip in the z-profile of the fluorescence intensity. Measurements were carried out in the upper (distal) cell membrane over 60–200 s. A series of 3–10 consecutive measurements was acquired at each position. After about every fifth measurement, the focal position was confirmed by recording a fluorescence profile. For assessing the effect of receptor stimulation on autocorrelation functions, EGF was added at a final concentration of 50 nM by carefully pipetting 200 μL of 250 nM stock solution to the 800 μL buffer already on the cells. Pairwise comparison of pre- and post-stimulus data are presented only for those experiments (~5% of total) where no significant displacement of the membrane with respect to the confocal detection volume took place during the series of measurements.

### 4.3. Evaluation of Autocorrelation Functions

Autocorrelation functions calculated on-line by the ALV-5000/E correlator board (ALV Laserbetriebsgesellschaft, Langen, Germany) were fitted assuming two different models.

(1) $$\mathrm{Model}\;1:\;G\left(\tau \right)=a_{0}+G_{triplet}\;G_{diff}^{free}$$

(2) $$\mathrm{Model}\;2:\;G\left(\tau \right)=a_{0}+G_{triplet,blink}\;G_{diff}^{anomal}$$

where

(3) Gtriplet(τ)=(1−T+Te−ττtr)1−T

(4) Gtriplet,blink(τ)=1−T−Θc+Te−ττtr+Θce−ττc1−T−Θc

(5) $$G_{diff}^{free}\left(\tau \right)=\frac{1}{N_{app}}\sum_{k=1}^{2}w_{k}{\left(1+\frac{\tau }{\tau_{k}}\right)}^{-1}{\left(1+\frac{1}{S^{2}}\frac{\tau }{\tau_{k}}\right)}^{-1/2}$$

(6) $$G_{diff}^{anomal}\left(\tau \right)=\frac{1}{N_{app}}\sum_{k=1}^{2}w_{k}{\left(1+{\left(\frac{\tau }{\tau_{k}}\right)}^{\alpha_{k}}\right)}^{-1}{\left(1+\frac{1}{S^{2}}{\left(\frac{\tau }{\tau_{k}}\right)}^{\alpha_{k}}\right)}^{-1/2}$$

(7) $$N_{app}=\frac{{\left(\sum_{k=1}^{2}B_{k}N_{k}\right)}^{2}}{\sum_{k=1}^{2}B_{k}^{2}N_{k}};\;w_{k}=\frac{B_{k}^{2}N_{k}}{N_{app}}$$

The autocorrelation function G(τ) can be broken down into terms accounting for triplet state formation (*G_triplet_*), dark state formation due to protonation (or light-induced transition to a non-emitting state) also called blinking (*G_triplet,blink_*), and terms accounting for diffusion (*G_diff_*). In the formula, *τ* is the lag time and *a*_0_ is an offset to compensate for the nonzero baseline of the autocorrelation function arising from a slow drift in the fluorescence signal (e.g., due to photobleaching). 

In the triplet term, *T* denotes the equilibrium molar fraction of fluorophores in the triplet state [49,50] and *τ_tr_* is the triplet correlation time. In [26], two independent protonation mechanisms of eGFP, an intramolecular proton transfer and a pH dependent external protonation process have been described. Since the characteristic time constants of the two protonation processes are separated by less than an order of magnitude at pH 7.4, a single term, characterized by the molecular fraction *Θ_c_* and the correlation time *τ_c_*, was considered. In the diffusion terms, which describe either free, unhindered diffusion $G_{diff}^{free}\left(\tau \right)$, or restricted, anomalous diffusion $G_{diff}^{anomal}\left(\tau \right)$, the diffusion of two species has been assumed: a fast one with a relative weight *w*_1_ and diffusion time *τ*_1_, and a slow one with a relative weight (1-*w*_1_) and diffusion time *τ*_2_. The weights depend linearly on the average number of molecules *N_k_* of the appropriate species in the detection volume and quadratically on their specific brightness *B_k_* (measured as signal arising from a molecule per unit of time). Because of the square dependence, oligomers with a higher brightness are overrepresented in the correlation function as compared to monomers. *S* denotes the axial ratio of the ellipsoid-shaped detection volume. Supposing that the slow diffusion component is entirely membrane-related, the square root term becomes unity (as if *S* were ∞). From a practical point of view, the obtained experimental values of *S*~7 are large enough to render this term to be unity. In the case of free diffusion (Equation (5)) the mean squared displacement is a linear function of time, whereas in the case of obstructed or anomalous diffusion (Equation (6)), this relation does not hold true; over a longer time, there is an increasing negative deviation from the linear relationship [27]. The anomaly parameter *α* is 1 for free diffusion and <1 for obstructed diffusion. *α*_1_ and *α*_2_ denote the anomaly parameters of the fast and slow components, respectively. Two models were tested: in model 1, free diffusion was assumed for both components (Equation (5)) and no blinking term was included, while for model 2, obstructed diffusion was allowed (leaving *α* to vary freely, Equation (6)) and blinking was also considered.

Weighted least squares fitting applying the Levenberg–Marquardt algorithm was performed using a program written in LabVIEW (National Instruments, Austin, TX, USA). The reciprocal of the variance of 5 residuals around each data point (differences between the actual data points and the values of the test function calculated with nearly optimal initial parameters) served as the statistical weight for the respective data point in the fit procedure.

The oligomerization state of receptors was assessed by calculating the apparent brightness from the total fluorescence intensity, *F*, and the apparent number of molecules, *N_app_*:

(8) $$\frac{F}{N_{app}}\propto \frac{\sum_{k=1}^{2}B_{k}^{2}N_{k}}{\sum_{k=1}^{2}B_{k}N_{k}}$$

*F*/*N_app_* is a weighted average of the brightness values of the different species, in which brighter species have a higher weight than their molar fractions because of the quadratic dependence on *B_k_*. Therefore, if the system is heterogeneous containing monomers and different oligomers, the apparent brightness is an overestimation of the average brightness. To correct for small variations in laser excitation intensity, *F*/*N_app_* was divided by the laser power *P*, assuming a linear dependence of intensity on power in the used range of light densities.

### 4.4. Background Correction

For cells expressing very few EGFR–eGFP fusion proteins (average number of molecules in the detection volume: *N* ≤ 1), background fluorescence was comparable to the average signal from the EGFR–eGFP molecules. In the presence of such a background, an aberrantly high number of molecules *N* is derived from the amplitude of the autocorrelation function. The background fluorescence was corrected for with the following formula [51]:

(9) $$G_{corr}\left(\tau \right)={\left(1-\frac{I_{B}}{I_{tot}}\right)}^{2}\left[G\left(\tau \right)-a_{0}\right]+a_{0}$$

*I_B_* is the uncorrelated background intensity (cellular autofluorescence and dark current of the photodiodes), and *I_tot_* is the total intensity including the signal and the background. G(τ) is any of the autocorrelation functions listed above and *a*_0_ is the offset term described in the previous sections.

### 4.5. Experimental Determination of the Dimensions of the Ellipsoid of the Confocal Detection Volume, and Calculation of the Diffusion Coefficient of EGFR–eGFP

For calculating the diffusion constant *D* from the diffusion time, knowledge of *ω_xy_* is necessary. To calibrate the size of the detection volume, the autocorrelation functions of a concentration series of fluorescein solutions (1, 2, 5, 10 nM) were measured [52]. Autocorrelations were fitted to the single-component free diffusion model including a triplet term, which yielded the number of molecules, *N*. For a single diffusing species, the average number of molecules in the detection volume can be expressed in terms of the dimensions of the e^−2^ ellipsoid in the optical plane and the molar concentration *c*:

(10) $$N=N_{A}\;c\pi^{3/2}\omega_{xy}^{2}\omega_{z}$$

where *N_A_* is Avogadro’s number. We measured the autocorrelation functions of fluorescein solutions at different dye concentrations ranging between 1 and 10 nM. The term $N_{A}\;\pi^{3/2}\omega_{xy}^{2}\omega_{z}$ was determined as the slope of the *N* vs. *c* plot. The axial radius of the ellipsoid, *ω_z_*, was determined experimentally by adsorbing ethidium bromide to the surface of a coverslip from a 10 μM solution of the dye. Fluorescence intensity was recorded, while the distance of the objective from the adsorbed dye layer was continuously changed by the piezoelectric focus positioner of the microscope. The intensity profile was fitted to a Lorentzian function:

(11) $$I\left(z\right)=I_{b}+\frac{2Aw}{\pi ({\left(4z-z_{0}\right)}^{2}+w^{2})}$$

where *I_b_* is the background intensity, *A* is a constant, *z* is the coordinate of the objective and *z*_0_ is the location of the peak. The width parameter *w* was used as an approximation for *ω_z_*. The value of *ω_xy_* was then calculated as the slope of the *N* vs. *c* plot divided by the term $\left(N_{A}\;\pi^{3/2}\omega_{z}\right)$. Typical dimensions for the setup used were *ω_xy_* = 0.34 ± 0.04 μm and *ω_z_* = 2.43 ± 0.58 μm, yielding an experimentally determined axial ratio *S* of ~7. 

Diffusion constants were then derived from the diffusional autocorrelation times according to the following formula:

(12) $$D=\frac{\omega_{xy}^{2}}{4\tau_{D}}$$

### 4.6. Statistical Analysis of FCS Data

To test the significance of changes in the fit parameters of autocorrelation functions upon stimulation by EGF and of the difference in the value of the anomaly parameters *α* from 1, two-tailed, paired t-tests were carried out.

### 4.7. Fluorescence Recovery after Photobleaching (FRAP)

F1-10 cells were plated into Nunc 8-well chambered coverglass plates (Thermo Fisher Scientific, Waltham, MA, USA) one day before the experiment. FRAP measurements were carried out on a Zeiss LSM 510 confocal microscope equipped with a 40× water immersion objective (NA 1.2). The 488 nm line of the Ar ion laser (1 kW/cm^2^) was used for exciting eGFP; emission between 505 and 550 nm was detected through a pinhole of 150 μm diameter (2.1 Airy units), resulting in an optical slice thickness of ~1.8 μm. Five 256 × 256 pixel pre-bleach images were scanned with a frame time of 498 ms and a digital resolution of 110 nm/pixel (8 × zoom). Bleaching was achieved by scanning a 10 pixel (1.1 μm) wide stripe across the shorter axis of the cell at 50 kW/cm^2^ laser power over ~700 ms. Subsequently, recovery of fluorescence was monitored for 50 s using the same settings as those used during the pre-bleach period. Stimulation with 50 nM EGF occurred as described for FCS measurements; cells before and 2.5 min after stimulation were measured. Mean fluorescence intensity in the bleached ROI of the cell was calculated using the LSM 510 software. Intensity vs. time traces were fitted by a smooth nonparametric curve to diminish noise. Mobility of EGFR–eGFP was assessed by determining the half recovery time T_1/2_ of fluorescence, by finding the time point corresponding to the midpoint between the intensity directly after bleaching and the final intensity at 50 s. 

Alternatively, FRAP curves were fitted to a bioexponential model function. First, fluorescence intensity vs. time curves were corrected for overall bleaching of the cell by dividing the intensity from the bleached ROI by the average intensity from the whole cell at each time point, and normalized to 1 by dividing by the average corrected pre-bleach intensity. Normalized curves were then separately averaged for control and for EGF-treated cells, and fitted to a two-component exponential function by using GraphPad Prism 8 (GraphPad Software, San Diego, CA, USA). The fast component, shared by the two curves, had a time constant of 1.7 s, which may be due to cytoplasmic receptors or, alternatively, to slow recovery from a light-induced dark state. The slow component was attributed to diffusion in the plasma membrane.

### 4.8. Western Blot Analysis of Tyrosine Phosphorylation after Receptor Stimulation with EGF

Tyrosine phosphorylation of cellular proteins was determined in Western blots of EGF-stimulated and control cell lysates. Aliquots containing lysates of 4 × 10^6^ cells were boiled in non-reducing SDS-PAGE sample buffer for 10 min. Proteins were separated electrophoretically on a Bio-Rad minigel apparatus (Bio-Rad, Richmond, VA, USA) using a 10% gel and were transferred to PVDF-Immobilon P membranes (Millipore, Burlington, MA, USA). Membranes blocked by Tween20-PBS (TPBS) containing 1% BSA were incubated overnight with 10 ng/mL sc-508-hrP peroxidase-conjugated anti-phosphotyrosine antibody (PY20, Santa Cruz Biochemicals, Santa Cruz, CA, USA) in TPBS-1% BSA. After washing three times in TPBS and once in PBS, membranes were developed with ECL reagents (Amersham Pharmacia Biotech, Piscataway, NJ, USA), exposed onto Fuji X-ray film and digitized at 1200 dpi. Total protein of the membrane was determined by amido-black staining.

### 4.9. Measurement of Ca^2+^ Responses upon EGF Stimulation

Intracellular Ca^2+^ concentrations were measured by fluorescence microscopy using the calcium indicator dye Fura-2 (Molecular Probes, Eugene, OR) [53]. F1-10, A431 and CHO cells were grown on 25 mm diameter round coverslips in DMEM. Before the experiment, cells were starved for 12 h in serum-free medium. Cells were loaded with 2 μg/mL Fura-2-AM for 30 min at 37 °C, washed twice with HBS (containing, in mM: 135 NaCl, 5 KCl, 1 MgCl_2_, 1.8 CaCl_2_, 5 glucose, 10 HEPES, pH 7.4) and imaged with an Attofluor Digital Ratio Imaging System (Atto Instruments, Rockville, MD, USA) with a time resolution of 0.2 frames per second. Spectral ranges were 340 ± 12.5 nm and 380 ± 12.5 nm for excitation, and larger than 520 nm for emission. After 100 s, 0, 8 or 50 nM EGF was administered, and at 400 s, 2 μg/mL of ionomycin (Sigma-Aldrich, St. Louis, MO, USA) was added to permeabilize the plasma membranes for Ca^2+^. To display activation-induced temporal changes in Ca^2+^ concentration, ratio images (I_334_/I_380_) corrected for field flatness were calculated, and the average ratios of regions-of-interest (ROIs) covering the inside of each cell were graphed versus time.

## 5. Conclusions

Membrane-localized EGF receptors have a high-mobility pool and a fast-bleaching, low-mobility pool. The fraction of the latter correlates positively with expression level, which is consistent with results recently shown by the Wohland group [54]. Upon ligand treatment, FCS and FRAP concurrently demonstrate the slowing down of EGFR on short (200 nm) and long (μm) distance scales, and the down-regulation of independently diffusing entities on the cell surface. Mobility is restored by 20 min after stimulation, but *N* remains lower, consistent with receptor internalization. The brightness of mobile receptors does not change in the first 5 min after stimulation, suggesting that the association of these mobile receptors with each other is not enhanced. Their slow-down indicates the association with slowly moving or immobile structures like the cytoskeleton or large, prebleached receptor aggregates.

## Figures and Tables

**Table 1:** Diffusion parameters of EGFR–eGFP before and 2 min after stimulation with 50 nM EGF.

FCS	Model 1 2-Component Free		Model 2 Blinking + 2-Comp. Anomalous	
	**Before EGF**	**After EGF**	**Before EGF**	**After EGF**
*D*_1_ (μm^2^/s)	24.9 ± 8.7 *	18.8 ± 9.1 *	19.7 ± 8.3	16.3 ± 9.4
*D*_2_ (μm^2^/s)	0.107 ± 0.037 *	0.057 ± 0.023 *	0.117 ± 0.051 *	0.066 ± 0.03 *
*w*_2_	0.77 ± 0.05	0.63 ± 0.12	0.70 ± 0.06	0.63 ± 0.12
*α*_1_	n.a.	n.a.	0.94 ± 0.17	0.82 ± 0.12 **
*α*_2_	n.a.	n.a.	0.99 ± 0.07	1.02 ± 0.08
*Θ_c_*	n.a.	n.a.	0.09 ± 0.01	0.09 ± 0.01
*τ_c_* (μs)	n.a.	n.a.	181 ± 21	187 ± 22
*T*	0.56 ± 0.11	0.58 ± 0.12	0.47 ± 0.08	0.51 ± 0.14
*τ_tr_* (μs)	1.7 ± 0.4	2.1 ± 0.8	2.3 ± 0.5	2.5 ± 0.9
mse	3.9	6.5	3.5	3.5
**Fluorescence recovery after photobleaching (FRAP)**	**Before EGF**	**After EGF**		
*D*_2_ (μm^2^/s) from *τ*_2_	0.067	0.040		
*w*_2_	0.64	0.69		
Immobile fraction	0.14	0.15		

Data are presented as means ± s. d. from ten cells, with autocorrelation recorded immediately before and after stimulation with 50 nM EGF. *D*: diffusion constant, *w*: weight of diffusion component, *α*: anomaly parameter, *T*: triplet fraction, *τ_tr_*: triplet correlation time, *Θ_c_*: fraction of dark species, *τ_c_*: correlation time of eGFP blinking, mse: average mean squared error of nonlinear fits. * Significant changes after EGF stimulation; *D*_1_, model 1: *p* = 0.017; *D*_2_, model 1: *p* = 0.023, model 2: *p* = 0.0016. ** *α*_1_ differs significantly from unity (*p* = 0.00004).

## Abbreviations

EGF	Epidermal growth factor
EGFR	Epidermal growth factor receptor
FCS	Fluorescence correlation spectroscopy
FRAP	Fluorescence recovery after photobleaching

## Appendix A

### Appendix A.1. Characterization of the Bleachable Fraction of EGFR Molecules

Fluorescence autocorrelation measurements were performed on cells expressing about 2 × 10^4^ of the EGFR–eGFP fusion proteins. When the laser excitation was switched on, even at low illumination intensity (~1 kW/cm^2^), a considerable fraction of the fluorescence decayed within several seconds, distorting the autocorrelation functions by mimicking the presence of a component with a long diffusion time. This decrease may be due to a combination of irreversible photobleaching and a reversible light-induced transition to a non-emitting state, limiting the analysis of diffusion rates and receptor numbers to molecules diffusing sufficiently fast to escape bleaching/blinking.

To address whether the bleached molecules were immobile, or they diffused too slowly to escape bleaching or whether the phenomenon was caused by a reversible light-induced transition, intermittent 15-s bleaching periods were applied with pauses of either 10–15 s or 50–200 s (Figure A1A). The count traces after longer pauses (third and sixth count traces) resembled the original bleaching curve (second count trace). Monitoring of the fluorescence intensity with a laser power of ~1 kW/cm^2^ before and after the bleaching intervals (1st and 5th count traces) showed that the total count rate decreased by only ~28% (from 62 to 45 kHz) over the first three 15-s bleaching periods (second, third and fourth count traces), whereas the transient decreases during the individual bleaching periods were much larger, at 85 ± 3%. This discrepancy between transient and total reductions in fluorescence showed that the majority of the bleachable molecules are mobile on a longer time scale, or that a substantial fraction of the observed decrease in intensity is caused by a reversible light-induced transition.

In order to avoid the continuous decrease in intensity during the autocorrelation measurements, the fluorescence of the fraction of molecules with low mobility was photobleached by illuminating the sample using an intensity of 10 kW/cm^2^ for 30–60 s, until the fluorescence was constant (see Figure 2A in the main text). The characteristic time constant of the decay was ~1–2 s. The remaining intensity was 26 ± 16% s. d. (*n* = 21) of the original one, i.e., the mobility of ~74% of the receptors was too low to escape photobleaching or light-driven blinking. The bleachable fraction correlated directly with the initial fluorescence intensity of the cell; the higher the initial intensity, the higher the bleachable fraction (Figure 2B in the main text).

In Figure A1A, count traces at 9 kW/cm^2^ and 0.9 kW/cm^2^ are shown. The fluorescence intensity at the lower laser power is much higher than 10% of the signal recorded at the higher laser power. This nonlinear dependence of emission on excitation intensity is indicative of the presence of photobleaching/light-induced blinking, which is more pronounced at a higher laser power, where considerable bleaching/blinking occurs even after reaching a steady signal level due to a dynamic equilibrium between photobleaching and re-population by diffusion.

Although the illuminated spot is confined to a relatively small fraction of the total cell surface, a decrease in the overall fluorescence of the cell was observed after completion of the FCS experiments. With typically 20 min of cumulative focal illumination at 1 kW/cm^2^ of power density, a decrease of ~20% in total cellular fluorescence was derived from wide-field fluorescence images, indicating that photobleaching is a major component of the observed decrease in intensity, and a significant fraction of the receptors has long-range mobility.

We also studied the dependence of apparent diffusion times on laser power. Photobleaching/blinking has a twofold effect on autocorrelation functions. First, the number of molecules in the detection volume is reduced. Second, photodestruction reduces the period during which a fluorophore emits photons in the detection volume, leading to an apparent decrease in the diffusional autocorrelation time [55,56] and thereby to an overestimation of the diffusion coefficient. The higher the excitation laser power, the stronger this effect. Figure A1B shows the correlation times as a function of the illumination intensity. The apparent correlation time of the fast component was barely affected by laser power (filled circles); the time spent in the illuminated volume was too short compared to the bleaching time constant. In contrast, the slow correlation time had a stronger negative correlation with the illumination intensity (open circles). To minimize this photobleaching effect, a low laser power still sufficient for recording autocorrelation functions with a good signal-to-noise ratio (~1 kW/cm^2^) was used in the FCS experiments.

### Appendix A.2. Effect of EGF Stimulation on F1-10 Cells—Individual Data

FCS parameters measured for individual cells immediately before and after stimulation by 50 nM EGF are shown in Figure A2. The change of the membrane diffusion coefficient *D*_2_ (Figure A2A), the change of the apparent number *N* of independently moving particles in the detection volume (Figure A2B), and the change of the normalized specific fluorescence per molecule (fpm, Figure A2C) are shown. *F* is the fluorescence intensity in kHz, *N* is the average number of molecules in the detection volume and *P* is the laser power. The ratio of specific brightness after stimulation to before stimulation is also displayed (Figure A2D). Apart from a few outliers, the value is around 1.
